# Transactions of the American Medical Association for 1856

**Published:** 1857-01

**Authors:** 


*Transactions of the American Medical Association for* 1856.

We had intended to notice this volume in the present number of the *Journal*, but we have only space left for the following letter. We hope all our readers will avail themselves of the opportunity to purchase a copy.

PHILA. Dec. 4.

Drs. PALMER & MILLER.

*Gentlemen*—Yours of Nov. 24th is at hand. You consent to charge yourselves with the sale of volumes of Transactions, and, in pursuance of the object, I have ordered twelve volumes for 1856 sent to your address. Will you be good enough to interest yourselves in this matter, as our funds will barely carry us through; and if the meeting next year is small we shall not be able to print the usual number of copies another year. The volumes I send are three dollars apiece to the profession. If you can dispose of more, I shall be happy to fill your orders.

Very respectfully yours, &c.

CASPAR WISTER,

*Treas. Am. Med. Assoc.* 479 *Arch St.*

